# Serotonin-related rodent models of early-life exposure relevant for neurodevelopmental vulnerability to psychiatric disorders

**DOI:** 10.1038/s41398-021-01388-6

**Published:** 2021-05-11

**Authors:** Tamara S. Adjimann, Carla V. Argañaraz, Mariano Soiza-Reilly

**Affiliations:** Instituto de Fisiología, Biología Molecular y Neurociencias (IFIBYNE), Consejo Nacional de Investigaciones Científicas y Técnicas (CONICET), Facultad de Ciencias Exactas y Naturales, Universidad de Buenos Aires, Buenos Aires, Argentina

**Keywords:** Neuroscience, Depression

## Abstract

Mental disorders including depression and anxiety are continuously rising their prevalence across the globe. Early-life experience of individuals emerges as a main risk factor contributing to the developmental vulnerability to psychiatric disorders. That is, perturbing environmental conditions during neurodevelopmental stages can have detrimental effects on adult mood and emotional responses. However, the possible maladaptive neural mechanisms contributing to such psychopathological phenomenon still remain poorly understood. In this review, we explore preclinical rodent models of developmental vulnerability to psychiatric disorders, focusing on the impact of early-life environmental perturbations on behavioral aspects relevant to stress-related and psychiatric disorders. We limit our analysis to well-established models in which alterations in the serotonin (5-HT) system appear to have a crucial role in the pathophysiological mechanisms. We analyze long-term behavioral outcomes produced by early-life exposures to stress and psychotropic drugs such as the selective 5-HT reuptake inhibitor (SSRI) antidepressants or the anticonvulsant valproic acid (VPA). We perform a comparative analysis, identifying differences and commonalities in the behavioral effects produced in these models. Furthermore, this review discusses recent advances on neurodevelopmental substrates engaged in these behavioral effects, emphasizing the possible existence of maladaptive mechanisms that could be shared by the different models.

## Introduction

Mental disorders including depression and anxiety are devastating and disabling conditions for the individual’s life, with an extremely concerning high prevalence worldwide. Currently, around 4.4% and 3.6% of the global population suffers from depressive and anxiety disorders, respectively, while the burden of mental illnesses is continuously rising^1,2^.

Psychiatric disorders arise from a complex combination of genetic, biological, environmental, and psychosocial factors. However, one of the main risk factors contributing to psychopathology corresponds to early-life adverse experiences during childhood, especially those involving traumatic or stressful events, such as maltreatment, abuse, poor or neglectful parenting, and loss of a family member, among others^3–7^. Furthermore, adverse experiences not only could contribute to pathological mental states, but may also affect the effectiveness of prospective therapeutic treatments^7,8^.

Brain imaging studies have shown that childhood maltreatment results in persistent alterations in a wide repertoire of neurocognitive systems involved in threat processing, reward, emotions, and executive control^6,8,9^. Interestingly, such alterations can often be found even in the absence of psychiatric conditions, indicating that maladaptive mechanisms produced by adverse environments in the early-life could predispose to latent vulnerabilities to future psychiatric disorders^8,9^.

Early-life exposure to certain drugs that interfere with the normal neurodevelopmental trajectories could also increase the predisposition of individuals to develop psychiatric disorders^10–13^. In particular, chemical substances that can interfere with crucial molecular and cellular neurodevelopmental events (e.g., cell proliferation, migration, differentiation, axon growth, synaptic connectivity, etc…) during the so-called critical periods^14–16^. These critical periods could represent unique developmental windows of brain plasticity, particularly relevant for preventive or therapeutic interventions^16–18^.

One example of this, is the use of antidepressant drugs that act as selective serotonin (5-HT) reuptake inhibitors (SSRIs) in pregnant and lactating depressed mothers^19,20^. These molecules have as a main molecular target the 5-HT transporter (SERT) typically present at the axon terminals of 5-HT neurons. Accumulating clinical evidence indicates that perinatal exposure to SSRIs could have a detrimental impact on infant’s neurodevelopment, with long-lasting consequences on cognitive and emotional capabilities^19–26^. In addition, other lines of evidence indicate that early exposure to SSRIs during pregnancy could increase the risk of Autism Spectrum Disorders (ASD)^27–32^, which in turn present a robust comorbidity with other psychiatric disorders including anxiety and depression^33,34^. However, there is a general agreement that adverse consequences observed after antenatal SSRI treatments should be always contrasted against the highly detrimental consequences of the unmedicated maternal mental illness.

To investigate the impact of early-life adverse experiences and exposure to drugs that could interfere with the normal neurodevelopment, several preclinical rodent models were developed, in which many of the emotional, social and cognitive aspects of human psychopathology can be recapitulated^10,12,35–40^. These models represent very useful tools to interrogate different aspects of the highly complex human pathophysiology, likely implying dysregulation of neurotransmitter systems, hormones and neuromodulators, growth factors, immune and inflammatory molecules, etc...^3,6,7,35,36^. In addition, brain structures such as prefrontal and sensory cortical regions, as well as other limbic regions including the amygdala, hippocampus and brainstem nuclei, have been increasingly driving the attention of clinicians and researchers as plausible neurodevelopmental substrates contributing to the vulnerability to mental illness^3,6,35,36,41^.

In this review, we explore well-established rodent models applied to study the impact of early-life environmental perturbations on adult behaviors of relevance to psychiatric disorders. We limited our analysis to models in which alterations in the 5-HT neurotransmission system appear to play a crucial role in the pathophysiological mechanisms^42–44^. Thus, we perform a comparative analysis across models of early-life exposures to stress and psychotropic drugs such as SSRIs or valproic acid (VPA), identifying differences and commonalities in their emotional and social behavioral outcomes, but also considering the impact of the perinatal period of exposure and treatment schedule on these effects. Importantly, this review discusses recent advances on developmental neural substrates engaged in such behavioral outcomes, also identifying possible maladaptive mechanisms likely shared by these models. To this aim, we carry out an exhaustive search in the PubMed database until February 2021, using a combination of the following key words: SSRI, fluoxetine, citalopram, serotonin, stress, early-life adversity, maternal separation, restrain, limited bedding/nesting, valproate, VPA, prenatal, gestational, pregnancy, postnatal, lactation, critical period, rodent, rat, mouse, emotional behavior, anxiety, depression, and mood. The results of this search were further refined and narrowed down to obtain only those studies that had sufficient methodological details to allow comparative analyses across the different models, as well as studies investigating the possible neurodevelopmental mechanisms contributing to the emotional and social behavioral effects.

## Early-life stress models: behavioral outcomes

In consonance with the primordial role of the early-life adverse experiences in the etiology of psychiatric disorders, several rodent models were established in which the dams and/or the offspring are exposed to different stressful stimuli during different neurodevelopmental stages. Importantly, in these models many of the emotional and cognitive impairments present in the human psychopathology can be consistently replicated^5,35,36^.

### **Gestational stress: restraint, unpredictable stress**

Two of the more frequently applied stressors to dams during the gestation are the restraint and unpredictable stress protocols. While the first one implies the physical body restraint of the dams in a closed compartment for time periods ranging from 1.5 to 6 h per day, in the second one, dams are daily subjected to a variety of different stressful stimuli including exposure to anxiogenic environments (social defeat paradigm), foot shocks (learned helplessness paradigm), forced swim, among others. Besides, these models vary according to whether the stress protocol is applied during the entire gestational period (i.e., until the offspring birth) or selectively during defined gestational windows, often resulting in distinctive behavioral outcomes.

Daily application of restraint or unpredictable stress protocols during almost the entire gestational period, in various rat and mouse strains, produces several stress-related and emotional alterations in the offspring later in life (Table 1). One of the most consistently reproduced phenotypes observed in these models, is the enhancement of anxiety behaviors, often accompanied by reduced locomotor and exploratory activities^45–49^. Additionally, these anxiety effects can predispose the offspring to other compulsive addictive behaviors like alcohol consumption^48^ (Table 1). Importantly, stress exposure in the late phase of the pregnancy, somewhere within the period from gestational day (G) 11 to postnatal day (P) 0, in mice and rats, has also produced robust anxiety phenotypes^50–57^ (Table 1).

**Table 1:** Early-life stress rodent models.

Stress type	Exposure period	Rodent model	Behavioral phenotypes	References
Restraint (6 h/day)	G5.5–G17.5	ICR mice	↓ Rearing and Locomotion (OF)	Miyagawa et al.^46,47^
			↓ Time and Entries in open arms (EPM)	
			↓ Rearing and Head dipping (Hole-board test)	
Restraint (2.25 h/day)	G7–P0	Swiss albino ND4 mice	↓ Time and Entries in open arms (EPM)	Dong et al.^48^
			↑ Time in dark compartment (Light-dark test)	
			↑ Alcohol consumption (2-bottle free choice)	
Restraint (2.25 h/day)	G12–G18	C57BL/6NCr mice	↓ Time in open arms (EPM)	Akatsu et al.^55^
			= No effects (MWM)	
Restraint (2.25 h/day)	G15–P0	Swiss albino mice	↑ Exploration and locomotion (T-maze, Tight-rope) (*in males*)	Pallarés et al.^51^
			↑ Entries in open arms (EPM) (*in females*)	
Restraint (1.5 h/day)	G5–G19	Wistar and Lewis rats NMRI and C57BL/6 mice	= No effects (OF)	Enayati et al.^49^
			↓ Sucrose preference (SPT) and ↑ Latency to feed (NSF)	
			↑ Immobility time (TST, FST)	
Restraint (2.25 h/day)	G14–G21	CD rats	↓ Entries in open arms (EPM) (*in females*)	Richardson et al.^50^
		Sprague-Dawley rats	↓ Time in open arms (EZM) (*in males*)	Van den Hove et al.^54^
Restraint (2.25 h/day)	G11–P0	Sprague-Dawley rats	↓ Time in the center (OF)	Zuena et al.^52^, Laloux et al.^53^
			↓ or ↑ Time in open arms (EPM) (*in males or females*)	
			↑ Spatial learning (MWM) (*in females*)	
Restraint (2.25 h/day)	G14–G21	Sprague-Dawley rats	↓ Time in the center (OF) (*in males*)	Iturra-Mena et al.^57^
	G15–G20		↓ Social interaction (*in both sexes*)	Poltyrev et al.^58^
			↓ Climbing and ↑ Immobility time (FST) (*in males*)	
			= No effects (SPT)	
Unpredictable chronic stress (1/day)	G1–G7	Wistar rats	= or ↑ Avoidance (ETM)	Soliani et al.^56^
	G8–G14		(*depending on exposure period*)	
	G15–G21			
Unpredictable chronic stress (1/day)	G13–G21	Wistar rats	↑ Swim velocity (MWM)	Barbie-Shoshani et al.^59^
			↑ Object recognition (*in females*)	
Unpredictable chronic stress (80 foot shocks/day)	G0–P0	Wistar rats	↓ Time and entries in open arms (EPM)	Estanislau and Morato^45^
Maternal separation (1 h/day)	P1–P11	C57BL/6 mice	↓ Flexibility (4 choice-reversal learning)	Thomas et al.^83^
Maternal and peer separation (4 h/day)	P2–P14	C57BL/6 mice	= No effects (OF)	Bailoo et al.^70^
Maternal separation	P2–P14	C57BL/6 mice	↓ Distance traveled and Rearings (*in males*) (OF)	Bondar et al.^65^
(4 h/day)			↓ Time in open arms (*in females*) (EPM)	
			↑ Social interactions (*in females*)	
Maternal separation (3 h/day)	P2–P14	C57BL/6 mice	↑ Time in the center and Rearings (OF)	Own and Patel^69^
			↓ Latency to first immobility (FST)	
Maternal separation (3 h/day)	P2–P14	BALB/cJRj	↓ Exploration and time in the center (OF)	Teissier et al.^67^
			↓ Time in open arms (EPM)	
			↑ Marble burying	
			↑ Immobility time (FST)	
			↓ Grooming time (Splash test)	
			↓ Short-term memory (Sequential novel object recognition)	
Maternal separation (4 h/day)	P2–P20	C57BL/6 mice	↑ Time and Entries in closed arms (EPM)	Shin et al.^79^
			= No effects (FST, Y-maze, MWM)	
			↑ Dominance (Tube test)	
			↓ Latency to first attack and ↑ Number of attacks (Resident intruder test)	
Maternal separation (1 h/day)	P3–P21	C57BL/6 mice	↑ Time in the center (OF) (after chronic social defeat stress)	Qin et al.^72^
			↑ Time and Entries in open arms (EPM) (after chronic social defeat stress)	
Maternal separation (4 h/day)	P10–P20	C57BL/6J mice	↓ Time in the center (OF) (after stress defeat)	Peña et al.^64^
			↑ Immobility time (FST) and ↓ Sucrose consumption (SPT) (after stress defeat)	
			↓ Social interaction (after stress defeat)	
Maternal separation		C57BL/6J and DBA/2 mice	↓ Time in the center (OF)	George et al.^61^
(4 h/day)	P2–P5			
(8 h/day)	P6-P16			
+ early weaning (at P17)				
			↓ Entries in open arms (EPM)	
			↑ Immobility time (FST) (*in DBA/2*)	
Maternal separation (6 h/day) + early weaning (at P17)	P7–P15	C57BL/6J mice	↑ Immobility time (TST)	Tchenio et al.^82^
			↓ Sucrose consumption (SPT)	
			↑ Failure to escapable shocks (Shuttle box)	
Maternal separation		Long Evans rats	↓ Freezing to conspecific	Litvin et al.^86^
(3 h/day)	P2–P13	↓ Unconditioned freezing (cat odor)		
(6 h/day)	P11-P13			
Maternal separation (3 h/day)	P1–P14	Wistar rats	= No effects (OF)	Farkas et al.^68^
Maternal separation	P2–P14	Sprague-Dawley rats	↓ Entries and Distance in the center (OF)	Benekareddy et al.^62,63^
(3 h/day)			↓ Entries and Distance in open arms (EPM)	
Maternal separation (3 h/day)	P2–P14	Long Evans rats	↑ Passive-submissive to proactive coping (Social Defeat)	Gardner et al.^85^
Maternal separation (3 h/day)	P2–P15	Wistar rats	↓ Time in open arms (EPM)	Uhelski and Fuchs^76^
Maternal separation (1,3 h/day)	P1–P14	Wistar-Kyoto (WKY)	↑ Exploration (*in WKY*) and ↓ (*in W*) (OF)	Rana et al.^71^
		and Wistar (W) rats	↓ Grooming (*in W*) (OF)	
			↓ Immobility time (FST) (*in WKY*)	
			↑ Social interaction (*in WKY*) and ↓ (*in W*)	
Maternal separation (6 h/day)	P2–P15	Wistar rats	↓ Time in the center (OF)	Roque et al.^77^
			↓ Latency to first immobility and ↑ Immobility time (FST)	
Maternal and peer separation (6 h/day)	P4–P14	Wistar rats	↓ Latency to enter the dark and ↑ Time in the dark (Light-dark test)	Kambali et al.^78^
			↓ Social novelty and ↑ Spatial learning (Radial arm maze)	
			↑ Attentional responses (5-Choice serial reaction time)	
Maternal separation (4 h/day)	P2–P20	Sprague-Dawley rats	↓ Social interactions and ↑ Latency to contact (nose-to-nose)	Holland et al.^84^
			↓ Distance to conspecifics (*in females*)	
Maternal separation (3 h/day)	P2–P21	Sprague-Dawley rats	↓ Distance in the center (OF)	Park et al.^66^
			↓ Time and Entries in open arms (EPM) and ↑ Immobility time (FST)	
Limited bedding and nesting material	P2–P9	C57BL/6J mice	= No effects (OF) and ↑ Latency to escape (MWM)	Rice et al.^73^
			↓ Novel object exploration	
Limited bedding and nesting material	P2–P9	C57BL/6 mice	= No effects (OF) and ↓ Time and Entries in bright compartment (Light-dark test)	Yang et al.^74^
			↓ Activity (Y-maze)	
Limited bedding and nesting material	P2–P9	C57BL/6J mice	= No effects (EPM, FST) and ↓ Object location memory	Naninck et al.^81^
			↓ Spatial learning (MWM) (*in males*)	
Limited bedding and nesting material	P4–P11	C57BL/6 mice	↑ Distance traveled (OF) (*in kicked pups*)	Gallo et al.^75^
			Time in bright compartment *(↑ in kicked and ↓ in non-kicked pups*) (Light-dark test)	
			= No effects (O-maze)	

In the studies where both sexes were analyzed, the sex-specific effects observed are indicated.

*OF* Open field, *EPM* Elevated plus maze, *EZM* Elevated Z-maze, *MWM* Morris water maze, *NSF* Novelty-suppressed feeding test, *TST* Tail suspension test, *FST* Forced-swim test, *SPT* Sucrose preference test, *ETM* Elevated T-maze.

Depressive-like symptoms were also reported after gestational exposure to stress in rats^49,58^, though the evidence is more limited (Table 1).

Examination of other behavioral components associated with psychiatric conditions, such as the social interaction to conspecifics, showed a marked reduction after prenatal stress^57^. In contrast, cognitive functions do not seem to be substantially affected in these models^55^, though moderate improvements in spatial learning^52,59^ and object recognition memory^59^, were observed in the offspring (Table 1).

### **Postnatal stress: maternal separation, limited beddingand nesting material**

Other models directly expose the offspring to the stressful stimuli. The repeated maternal separation of the pups from the dams has been consistently used as an efficient stressful condition, especially when it happens during the lactation period^60^. This model sometimes also includes other stressors like the early weaning of the pups or a limited access to the bedding and nesting materials.

Protocols of maternal separation vary according to the time the pups spend isolated from their dams, but also to during which postnatal period the protocol is applied. Thus, while the time of separation typically ranges from 1 to 8 h per day, the postnatal period usually covers the first 2 or 3 postnatal weeks. In addition, in these models, distinctive behavioral effects can be observed when considering the mouse/rat strain and the sex of the offspring.

Daily separation from the dams during the first 2 or 3 postnatal weeks, in various mouse/rat strains, have consistently produced a reduction in the exploratory activity of the offspring^61–67^. Besides, these effects appeared to be more commonly detected in males than in female littermates^65^. However, other studies using slightly different conditions, could not reproduce these findings^68–72^. A combination of maternal separation with an early weaning of the pups also produced a decreased exploration^61^. On the other hand, stress protocols of limited access to bedding and nesting material, were shown to be inefficient per se to reproduce the exploratory defects^73–75^ (Table 1).

Anxiety phenotypes were consistently observed in various mouse/rat strains after maternal separation during the first 2^62,63,65,67,71,76–78^ or 3 postnatal weeks^61,64,66,79,80^ (Table 1). However, milder protocols (1 h/day) appeared to be insufficient to produce anxiety effects, after a chronic social distress^72^. On the other hand, anxiety phenotypes were reported to be more evident in females than in male descendants^65^. Lastly, less robust anxiety phenotypes were observed when both the dams and pups had a limited access to the bedding and nesting material during early postnatal life^74,75,81^ (Table 1).

Depressive-like behaviors have been repeatedly observed after maternal separation protocols in various mouse strains^64,67,69^, though others failed to reproduce these effects^79^. Similar depressive-like symptoms were reported when the maternal separation was followed by an early weaning in mice^61,82^. Interestingly, a mouse study suggested that the maternal separation from P10 to P20 would be sufficient to induce depressive-like symptoms in the offspring^64^. In rats, depressive-like phenotypes produced by maternal separation protocols are less robust. Thus, depressive-like symptoms were reported in Sprague-Dawley and Wistar rats^66,77^, while other studies using briefer protocols (1.2 h/day) in Wistar-Kyoto rats, have described the opposite effects^71^ (Table 1).

The impact of early maternal separation upon cognitive behaviors was consistently evidenced (Table 1). In general, postnatal exposures to stress appeared to have more profound consequences on cognitive abilities than prenatal exposures. Studies carried out in various mouse strains have shown deficits in behavioral flexibility^83^ and short-term memory^67^, after different maternal separation protocols. In contrast, no apparent effects were observed on spatial learning and working memory in mice^79^. However, in rats, improvements in spatial learning and attention were recently described^78^. Detrimental consequences of the limited bedding and nesting material have been observed on the novel object exploration and location memory^73,81^, accompanied by deficits in spatial learning and working memory tasks^73,74,81^ (Table 1).

Consequences of maternal separation on social behaviors were reported in mice and rats (Table 1). Thus, a delayed latency to initiate social contacts, and a decreased engagement in social interactions and activities, have been found^64,71,78,84^. However, other studies could not reproduce some of these effects^65^. Interestingly, distinct behavioral features associated with social behaviors, like aggression or dominance, were also found to be enhanced by maternal separation^79,85^. Consistent with this, a reduction of freezing responses to conspecifics, or after a punishment, were also observed^86^ (Table 1).

## Pharmacological models: behavioral outcomes

Psychotropic drugs can interfere directly or indirectly with the brain’s developmental trajectory. Importantly, the age period when such perturbations occur will define the emergence of long-lasting detrimental consequences on the individual’s brain architecture and function. Rodent models have been very useful to understand how such dysregulation of neural mechanisms during developmental critical periods can impact adult behaviors. Here, we analyze the behavioral outcomes of two pharmacological models: the exposure to SSRI antidepressants, and to the anticonvulsant and mood stabilizer, VPA. Both drugs have a high capacity of crossing the placenta to reach the fetus^10,87,88^. While SSRIs inhibit the reuptake of 5-HT from the extracellular space by blocking the 5-HT transporter, SERT, the precise mechanism of action of VPA is less known. In fact, it is thought that one of the main actions of VPA is to modulate the synthesis and metabolism of gamma-aminobutyric acid (GABA), resulting in an enhancement of GABA neurotransmission^87^. Besides, VPA was also shown to have a direct inhibitory action on histone deacetylases (HDACs), modulating the epigenetic control of gene expression^89^.

### **Gestational exposure to SSRIs**

One of the most widely prescribed SSRI antidepressants is fluoxetine, and its exposure during the gestational period has a neurodevelopmental impact in the offspring (Table 2), as indicated by a recent meta-analysis by Ramsteijn et al.^90^. Rat pups prenatally exposed to fluoxetine showed a mild delayed neurological maturation^91^, followed by a general reduction in exploratory behaviors, accompanied by anxiety and depressive-like phenotypes^92,93^. On the other hand, studies applying a much broader schedule of fluoxetine treatment, covering from the last phase of the gestation to somewhere until the third postnatal week, could not detect such exploratory and emotional deficits^94–98^, while including the full gestational period produced emotional modifications^99^ (Table 2).

**Table 2:** Rodent models of early-life exposure to SSRIs.

SSRI (daily dose)	Exposure period	Rodent model	Behavioral phenotypes	References
Fluoxetine (0.3–0.8 mg/kg i.p.)	G8–G18	C57BL/6J mice	↓ Distance traveled in the center (OF)	Noorlander et al.^92^, Smit-Rigter et al.^93^
			↑ Time in closed arms (EPM)	
			↑ Latency to feed (NSF)	
Fluoxetine (10 mg/kg s.c.)	G1–P0	CD1 mice	= No effects (Novel object exploration, Object memory test)	Svirsky et al.^100^
			↑ Animal exploration (Social preference test) (only in young females)	
			= No effects (Social exploration and Social Memory tests)	
			↑ Number and Duration of attacks (Social exploration and memory tests)	
Fluoxetine (25 mg/kg per os.)	G15–P12	C57BL/6 mice	= No effects (OF)	Kiryanova et al.^95,96^
			↑ Time in open arms and Number of head dips (EPM)	
			↑ Spatial memory (MWM)	
			= No effects (Passive avoidance, PPI)	
			↑ Proportion of attackers	
			= No effects (Social interaction) (Resident intruder)	
Fluoxetine (25 mg/kg per os.)	G15–P12	C57BL/6 mice	= No effects (OF, Horizontal ladder, PPI, MWM, Fear conditioning)	McAllister et al.^94^
			↓ Time in closed arms (EPM)	
			↑ Latency to first immobility (FST)	
Fluoxetine (10 mg/kg per os.)	G0–P14	Sprague-Dawley and Wistar-Kyoto rats	↓ Time in the center (OF)	Millard et al.^99^
			↓ Time in open arms (EPM)	
			↑ Immobility time (FST)	
Fluoxetine (10 mg/kg per gavage)	G0–P21	Wistar rats	↓ Third-party prosocial behavior (in females)	Heinla et al.^102^
Fluoxetine (5 mg/kg per gavage)	G1–P21	Wistar rats	= No effects (OF, EPM)	Toffoli et al.^98^
Fluoxetine (10 mg/kg per os.)	G10–P21	Sprague-Dawley rats	*In females:*	Gemmel et al.^101^
			↑ Time interacting with another female	
			↑ Time in social investigation	
			↓ Time to first interaction	
			*In males:*	
			↑ Time in social play	
			↑ Running away from a novel partner	
			↑ Self grooming	
Fluoxetine (12 mg/kg per gavage)	G11–P0	Wistar rats	= No effects (OF, EPM, SPT, FST)	Olivier et al.^97^
			↑ Latency to feed (NSF)	
			↑ Freezing and ↓ Time in the shock compartment	
			(Place aversion)	
			↓ Juvenile social play and exploration	
			↓ Adult self-grooming and social exploration	
Fluoxetine (12 mg/kg per gavage)	G11–P7	Wistar rats	↑ Turning time (Negative geotaxis)	Kroeze et al.^91^
			↑ Age (Vibrissa placement)	
			↑ Age (Startle reflex)	
			↓ Locomotor and motor abilities (at early postnatal ages)	
			↓ Grooming	
			= No effects (NOR, Object directed behavior)	
Fluoxetine (10 mg/kg i.p.)	P4–P21	129S6/SvEvTac mice	**↓** Distance traveled, Rearing and Ambulation times (OF)	Ansorge et al.^103^
			↓ Number of open arm entries (EPM)	
			↑ Latency to escape (shock-avoidance/escape)	
			↑ Latency to feed (NSF)	
Fluoxetine (10 mg/kg i.p.)	P2–P11	129S6/SvEvTac mice	↓ Sucrose consumption (SPT)	Rebello et al.^106^, Teissier et al.^107^
			↑ Immobility time (FST)	
Fluoxetine (10 mg/kg per os.)	P2–P14	C57BL/6J mice	↑ Latency to feed (NSF)	Soiza-Reilly et al.^111^
			↑ Immobility time (FST)	
Fluoxetine (10 mg/kg s.c.)	P2–P14	C57BL/6J mice	↓ Time and Total distance in the center (OF)	Olusakin et al.^112^
			↑ Latency to feed (NSF)	
			↑ Immobility time (FST)	
			↑ Latency to groom (Splash test)	
Fluoxetine (5 mg/kg per os.)	P1–P21	C57BL/6J mice	= No effects (MWM)	Ishiwata et al.^119^
Fluoxetine (10 mg/kg i.p.)	P4–P21	C57BL/6J mice	↑ Immobility time in the center, ↓ Total rearing time (OF)	Karpova et al.^113^
			↑ Total immobility time (Light-Dark)	
			↓ Immobility time (FST)	
Fluoxetine (5 mg/kg per os.)	P1–P21	BALB/c mice	↑ Time and Entries in open arms (EPM)	Ishikawa and Shiga^116^
			= No effects (FST, SPT, MWM)	
Fluoxetine (10 mg/kg s.c.)	P0–P6	Wistar rats	↓ Maximum crossable gap distance	Lee et al.^117^
			(Gap-crossing)	
			↓ Ambulation in the center and rearing (OF)	
Fluoxetine (20 mg/kg s.c.)	P0–P4	Wistar rats	↓ Distance traveled and ambulation in the center (OF)	Ko et al.^115^
			↓ Number of closed arm entries and of total distance traveled (EPM)	
			↑ Time spent in immobility (FST)	
			↓ Sensorimotor gating (PPI)	
			↑ Social interaction, sniffing and contacts	
Fluoxetine (10 mg/kg per gavage)	P2–P7	NIH Norway rats	↓ Ultrasonic vocalizations (PPI)	Zimmerberg and Germeyan^118^
			↓ Interaction time with conspecific	
Fluoxetine (5 mg/kg s.c. osmotic minipump in dams)	P1–P21	Sprague-Dawley rats	*In females:*	Boulle et al.^109,110^
			= No effects (OF, EZM)	
			↑ Immobility time (FST)	
			*In males:*	
			↓ Center entries (OF)	
			↓ Distance traveled (EZM)	
			= No effects (FST)	
Fluoxetine (10 mg/kg s.c.)	P1–P21	Wistar rats	↓ Time in closed arms	Da Silva et al.^114^
			↑ Number of open arm entries and Time in open arms (EPM)	
Fluoxetine (10 mg/kg per os.)	P2–P21	Sprague-Dawley rats	↓ Time and Traveled distance in the center (OF)	Sarkar et al.^104,105^
			↓ Path length and time in open arms (EPM)	
			↑ Immobility time (FST)	
			↓ Juvenile play behavior and Time in social grooming	
Fluoxetine (10 mg/kg i.p. in dams)	P2–P24	Sprague-Dawley rats	↑ Time in closed arms (EPM) (*in males*)	Gobinath et al.^108^
			↑ Latency to feed (NSF) (*in males*)	
			↑ Swim time (FST)	
Citalopram (10 mg/kg i.p.)	P4–P21	129S6/SvEv mice	↓ Total ambulatory time (OF)	Ansorge et al.^121^
			↓ Total number of arm entries (EPM)	
			↑ Latency to escape (shock-escape)	
			↑ Latency to drink (novelty-induced hypophagia)	
Citalopram (20 mg/kg s.c.)	P1–P10	Sprague-Dawley rats	↑ Auditory discrimination threshold (*in males*)	Zhou et al.^214^
Citalopram (10 mg/kg s.c.)	P8–P21	Long Evans rats	↑ Distance traveled and ambulatory time (OF)	Maciag et al.^122,123^, Harris et al.^124^
			= No effects (EPM, FST, Saccharine-sweetened fluid consumption)	
Citalopram (20 mg/kg s.c.)	P8–P21	Long Evans rats	↑ Stereotypic behaviors and Freezing after tone (*stronger in males*)	Simpson et al. [126], Rodriguez-Porcel et al.^127^ Khatri et al.^128^
			↓ Exploration of novel object (*stronger in males*)	
			↓ Juvenile play behavior and interaction to conspecifics (*stronger in males*)	
Escitalopram (10 mg/kg s.c.)	P5–P19	CD1 mice	↓ Total arm entries and rears (EPM)	Popa et al.^120^
			↓ Sucrose consumption and intake (SPT)	
			↑ Immobility time (FST and TST)	
			↑ REM sleep (pro-depressive)	
			= No effects (light-dark)	
Escitalopram (10 mg/kg s.c.)	P5–P21	CD1 × 129SvEv mice	↑ Time and Entries in Open arms (EPM)	Altieri et al.^125^
			= No effects (OF, FST)	

In the studies where both sexes were analyzed, the sex-specific effects observed are indicated.

*OF* Open field, *EPM* Elevated plus maze, *PPI* Prepulse inhibition, *MWM* Morris water maze, *NSF* Novelty-suppressed feeding test, *TST* Tail suspension test, *FST* Forced-swim test, *SPT* Sucrose preference test, *EZM* Elevated Z-maze.

Regarding cognitive and social behaviors, only moderate effects of gestational exposures to SSRIs were observed (Table 2). Thus, no major changes in the exploration or memory of novel objects were detected in mice and rats^91,100^. However, these studies did observe increments in conspecific exploration and aggression^100^. Consistently, an increased proportion of “attacker” individuals was found after fluoxetine exposure at later perinatal periods (G15 to P12)^95,96^. In the same conditions, no major effects on passive avoidance, fear conditioning, spatial learning and prepulse inhibition (PPI) were detected in mice^94,96^. On the other hand, in rats, the freezing behavior was shown to be enhanced by prenatal fluoxetine treatment, and several sex-specific social outcomes were also described^97,101,102^ (Table 2).

### **Postnatal exposure to SSRIs**

Other studies investigated the behavioral consequences of exposures to SSRIs during the early postnatal period^10,12,103^ (Table 2). Fluoxetine application during the first 2 or 3 postnatal weeks in various mouse/rat strains resulted in a marked reduction of exploratory behaviors, accompanied by the emergence of anxiety and depressive-like phenotypes^103–112^. Others, applying similar protocols of fluoxetine exposure, only partially reproduced these emotional effects^113–116^ (Table 2).

Other behavioral effects of relevance for psychiatric conditions were also reported after postnatal exposure to SSRIs. That is, several rat studies have reported reductions in ultrasonic vocalizations and sensorimotor responses, accompanied by alterations in social interactions to conspecifics^104,105,115,117,118^. On the other hand, spatial learning and memory abilities seemed not to be affected by the fluoxetine treatment at lower doses^116,119^ (Table 2), consistent with a recent meta-analysis^90^.

Postnatal exposure to other SSRIs like citalopram or escitalopram, during the same postnatal period, also produces anxiety and depressive-like phenotypes in mice^120,121^. However, other studies have only partially replicated some of these emotional effects^122–125^. In addition, citalopram exposure in the same period, was shown to reduce the exploration of novel objects and the engagement in juvenile play, besides of exacerbating the freezing response to a tone^126–128^ (Table 2).

Since SSRIs are often prescribed to depressed pregnant women, preclinical investigations on the possible interaction of these treatments with the maternal stress become highly relevant for obvious direct translational reasons. Several studies have shown that early postnatal fluoxetine treatment in pups exposed to either prenatal stress or maternal separation, can alleviate anxiety and depressive-like symptoms produced in these models^110,129–131^. However, this does not seem to be the case when using another SSRI (i.e., citalopram)^132^.

### **Gestational and postnatal exposures to VPA**

Acute treatment with a high dose of VPA during pregnancy causes a wide repertoire of emotional, social and cognitive alterations in the offspring (Table 3). Although the exact molecular mechanism of action of this drug remains unknown, VPA has been consistently applied during neurodevelopment in an attempt to replicate common phenotypic features present in ASD patients^39,40^.

**Table 3:** Rodent models of VPA exposure.

VPA dose	Exposure period	Rodent model	Behavioral phenotypes	References
500 mg/kg i.p.	G9, G12.5, or G14.5	ICR(CD1) mice	↓ Distance and Entries in the center (OF) (*for G12.5, in both sexes*)	Kataoka et al.^137^
			↓ Time in open arms (EPM) (*for G12.5, in both sexes*)	
			↓ Social interaction (Sniffing) (for G12.5, in males)	
			↑ Social interaction (Sniffing) (*for G12.5, in females*)	
			↓ Spatial learning (MWM) (*for G12.5*)	
800 mg/kg per os.	G11	Hybrid mice (C57BL/6, CF-1, Swiss, DBA/2)	↑ Latency to reach home bedding	Roullet et al.^158^
			↓ Social behavior (Nose pokes)	
			↓ Social novelty (Nose pokes)	
200 mg/kg s.c.	G12–G17	BALB/c mice	= No major effects (Negative geotaxis, Surface righting, Balance beam).	Wagner et al.^133^
			↑ Delay (Surface and Mid-air righting) (*in both sexes*)	
600 mg/kg s.c.	G13		↓ Latency to fall (Grip strength) (*in both sexes*)	
			↑ Locomotor activity	
			↓ Spatial learning (MWM) (*in both sexes*)	
600 mg/kg s.c.	G12.5	Crl:Fcen:CF1 mice	↓ Juvenile play (Solicitations, sniffing, Following)	Campolongo et al.^147^, Acosta et al.^167^, Kazlaukas et al.^162^
			↑ Self-grooming	
			↓ Alternation and distance (Y-maze, EPM, OF)	
			= No effects (Affiliative and Non-social behaviors, Odor habituation, NOR, Light-dark test)	
			↓ Social preference and interaction (Sniffing) (*in males*)	
			↑ Immobility time (TST, FST)	
			↓ Temporal Accuracy and Precision (Interval timing)	
500 mg/kg i.p.	G12.5	C57BL/6J mice	↑ Marble burying	Wu et al.^164^
			↓ Social interaction (time)	
			↓ Social preference (time)	
			↓ Learning (Negative Reinforcement Task)	
600 mg/kg i.p.	G12.5	C57BL/6J mice	↑ Grooming and Digging time	Moldrich et al.^145^
			↓ Social interaction (time, nose pokes, approaches)	
			↓ Olfactory motivation	
600 mg/kg s.c.	G13	C57BL/6Hsd mice	↓ Entries and Time in the center (OF)	Mehta et al.^138^
			↑ Self-grooming and Marble burying	
800 mg/kg per os.	G9	Wistar rats	↓ Time of all pellet consumption and Exploration (Radial maze)	Narita et al.^136^
			↑ Locomotor activity without anxiety effects (OF)	
			= No effects (Social interaction)	
600 mg/kg i.p.	G9	Wistar rats	↑ Time spent in empty compartment, ↑ Crossings between social compartments, ↓ Initiation of social approaches	Dufour-Rainfray et al.^149^
800 mg/kg per gavage	G9	Wistar rats	↑ Locomotor activity (OF) (in light/sleep phase)	Tsujino et al.^141^
			↑ Feeding (in light/sleep phase)	
500 mg/kg i.p.	G11.5	Wistar Han rats	↑ Entries to the same arm (Y-maze)	Favre et al.^160^
			↓ Social preference (sniffing)	
			↑ Freezing (Pavlovian fear conditioning)	
600 mg/kg i.p.	G12.5	Wistar rats	↑ Latency to reach home bedding (*olfactory discrimination in pups*)	Schneider and Przewłocki^134^, Schneider et al.^139,140,152^
			↓ Angle of swim (*in pups*)	
			↑ Locomotor and Stereotypic behavior (*in both sexes*)	
			↓ Exploratory activity (Rearing and hole poking)	
			↓ Entries and Time in open arms (EPM) (*in males*)	
			↓ Sensorimotor gating (PPI)	
			↓ Social play and Social exploration behavior (*in males*)	
			= No effects (NOR)	
500 mg/kg i.p.	G12.5	Wistar Han rats	↑ Entries to the same arm (Y-maze)	Markram et al.^142^
			↓ Time in open arms (EPM)	
			↓ Social interaction (sniffing, touching)	
			↓ Sensorimotor gating (PPI)	
			↑ Tone and Context memories, Generalization and Extinction (Fear conditioning)	
			= No effects (Locomotion, MWM)	
500 mg/kg i.p.	G12.5	Wistar rats	↑ Time in closed arms (EPM) (*in both sexes*)	Edalatmanesh et al.^146^
			↑ Repetitive behavior and ↓ Alternation behavior (Y-maze) (*in both sexes*)	
			↓ Play behavior, Social exploration and contact (*in both sexes*)	
			↑ Spatial learning and memory (MWM) (*in both sexes*)	
400 mg/kg s.c.	G12.5	Wistar rats	↓ Time in open arms (EPM)	Ellenbroek et al.^155^
			↑ Latency to feed (NSF)	
			↑ Sucrose consumption (Latent inhibition)	
			↓ Sensorimotor gating (PPI)	
600 mg/kg i.p.	G12.5	Wistar rats	↓ Social exploration and preference	Bambini-Junior et al.^143^
			↑ Alternation delay (Y-maze)	
			= No effects (MWM)	
600 mg/kg i.p.	G12.5	Wistar rats	↓ Time in the center (OF) (*in both sexes*)	Olexová et al.^156^
400 mg/kg s.c.	G7, G9.5, G12 or G15	Sprague-Dawley rats	↓ Interaction to familiar and novel conspecifics (*more robust for G12 exposure, in males*)	Kim et al.^150,159^
			↓ Social preference (*in males*)	
500 mg/kg per gavage	G11–13	Sprague-Dawley rats	↓ Ultrasonic vocalizations (*in both sexes*)	Barrett et al.^157^
			↓ Time in the center (OF) (*in females*)	
			↑ Baseline startle amplitude (*in males*)	
			↑ Startle response (after Fear conditioning) (*in both sexes*)	
			↓ Approach to a social stimulus (*in both sexes*)	
			↓ Novel social interaction (*in males*)	
600 mg/kg i.p.	G12	Sprague-Dawley rats	↑ Freezing (Trace and Delay Fear conditioning)	Sui and Chen^165^
500 mg/kg i.p.	G12.5	Sprague-Dawley rats	↓ Time in the center (OF)	Lin et al.^153^
			↓ Time in open arms (EPM)	
			↓ Social interaction (duration and frequency)	
			↑ Freezing (Contextual fear conditioning)	
500 mg/kg i.p.	G12.5	Sprague-Dawley rats	↓ Ultrasonic vocalizations (*in both sexes*)	Gzielo et al.^163^
600 mg/kg i.p.	G12.5	Sprague-Dawley rats	↑ Delays in Surface and Air righting reflexes, Negative geotaxis, Cliff aversion, Crawling and Visual placing reflex	Hou et al.^135^
			↓ Motor abilities (Swimming, Front limb suspension)	
			↑ Onset of auditory startle	
			↑ Self-grooming	
			↓ Center entries (OF)	
			↓ Social preference and interaction	
			↓ Novel social interaction	
			↓ Spatial learning (MWM)	
500–600 mg/kg i.p.	G12.5	Sprague-Dawley rats	↑ Freezing (Fear conditioning)	Wang et al.^166^, Banerjee et al.^154^
			↓ Entries and Distance traveled in the center (OF)	
			↓ Social interaction and Social visits	
			= No effects (NOR)	
800 mg/kg per os.	G12	Long Evans rats	↓ Performance (T-maze) (*in both sexes*)	Mychasiuk et al.^144^
			↑ Time in open arms (EPM) (*in both sexes*)	
			↑ Interaction to familiar object (NOR) (*in both sexes*)	
			↑ Performance (Whishaw tray reaching test) (*in males*)	
			↓ Performance (Whishaw tray reaching test) (*in females*)	
600 mg/kg i.p.	G12	Long Evans rats	↓ Sniffing (*in females*)	McKinnell et al.^148^
			↑ Self-grooming (*in both sexes*)	
			↑ Interaction to familiar object (NOR) (*in males*)	
			↓ Marble burying	
			↓ Performance (Set shifting task) (*in both sexes, stronger effects in females*)	
800 mg/kg per os.	G12.5	Long Evans rats (females)	↓ Defensive rotation tactic and ↑ Standing tactic	Raza et al.^161^
			↑ Frequency of Mounting, Head and Body shaking	
			↓ Ultrasonic vocalizations (at Social play)	
			= No effects (Playful attacks)	
350 mg/kg i.p.	G13	Long Evans rats	↑ Social exploration and Play fighting (*in adolescence*)	Cohen et al.^151^
200–400 mg/kg s.c.	P14	BALB/c mice	= No effects (Grip strength, Balance beam, Locomotor activity) and ↑ Delay in Negative geotaxis and Mid-air righting	Wagner et al.^133^
			↓ Spatial learning (MWM) and ↑ Latency (Passive avoidance)	
400 mg/kg s.c.	P14	BALB/c mice	↓ Social behaviors (allogrooming, crawl under/over, sniffing)	Yochum et al.^170^
			↓ Motor activity (in social environment)	
			↑ Locomotor activity	
400 mg/kg s.c.	P14	C57BL/6J mice	↑ Time in open arms (EPM) (*only in males*)	Norton et al.^169^
			↓ Reversal learning (Water Y-maze)	
			↑ Social aggressions (*only in males*)	
			= No effects (Locomotion, Social approach, PPI, Allogrooming, Sniffing)	
300 mg/kg s.c.	P2–P4	Sprague-Dawley rats	↑ Exploration (OF) and ↓ Entries and Time in open arms (EPM)	Mony et al.^171^
(twice/day on P2–P3 and once at P4)				
			↓ Social preference and interaction (Ttime spent, Sniffing, Grooming, Mounting, Crawling)	
			= No effects (Passive avoidance)	
150 mg/kg/day i.p.	P6–P20	Sprague-Dawley rats	↑ Delay in eye opening	Chomiak et al.^168^
			↓ Social play (rough-and-tumble)	
			↑ Cue-dependent reward learning	

In the studies where both sexes were analyzed, the sex-specific effects observed are indicated.

*OF* Open field, *EPM* Elevated plus maze, *PPI* Prepulse inhibition, *MWM* Morris water maze, *NSF* Novelty-suppressed feeding test, *TST* Tail suspension test, *FST* Forced-swim test, *NOR* Novel object recognition.

Most of the studies reporting behavioral effects in various mouse/rat strains use a single dose of VPA (400–800 mg/kg) in a given day, within the period G11–G13 (Table 3). In these conditions, the exposed-offspring presents substantial neurological maturation delays^133–135^, usually accompanied by a reduced exploratory activity^136–138^, and the enhancement of self-grooming and stereotypic behaviors^133–136,138–148^. This is highly consistent with the exacerbation of repetitive behaviors observed in ASD patients^39,40^. Interestingly, long-lasting deficits in motor and procedural skills were also observed after VPA exposure^144^. On the contrary, neither lower doses nor VPA exposures before or after such gestational period, lead to robust behavioral outcomes^133,136,137,141,149–151^ (Table 3).

Anxiety phenotypes induced by VPA exposure during the pregnancy were more consistently found in rats than in mice^135,137–139,142,146,147,152–157^. However, other studies could not replicate some of these effects^144^. On the other hand, the emergence of depressive-like symptoms was also reported in a mouse study^147^ (Table 3).

Social behaviors appeared to be markedly affected by prenatal VPA in various mouse/rat strains, indicating a highly consistent reduction in the number of ultrasonic vocalizations and social interactions (e.g., nose pokes, approaches, etc…), accompanied by a diminished juvenile social play, and limited interest for novel conspecifics^134,135,139,140,142,143,145–147,149,150,153,154,157–164^. However, several studies failed to replicate some of these effects^136,137,151^. On the other hand, and related to the social deficits observed, a decreased olfactory motivation and sensorimotor capacity were found in VPA-exposed mice^134,139,142,145,155^ (Table 3). Additionally, sex-specific aggressive and defensive strategies in social settings appeared to be affected by the VPA exposure, switching from a defensive tactic to a more aggressive one^161^. Furthermore, enhanced freezing and startle responses were described in different fear-conditioning paradigms after VPA treatment^142,153,154,157,160,165,166^ (Table 3).

Certain cognitive aspects have been described to be altered in VPA gestational models, though the behavioral outcomes were less consistent. Thus, several studies reported either deficits^133,135,137^ or improvements in spatial learning^146^, while others could not reproduce these effects^142,143^. Similarly, evaluation of exploration time of a novel object showed that VPA treatment increased the interaction to familiar objects^144,148^, though other researchers could not replicate these findings^147,152,154^. Additionally, deficits in working memory^146,147^, temporal accuracy^167^, attentional, and negative reinforcement learning tasks^148,164^ were also reported (Table 3).

There are fewer studies applying postnatal exposures to VPA, having in general, less robust behavioral effects (Table 3). VPA treatment somewhere during the first 2 postnatal weeks produces a delayed neurological maturation^133,168^, without any apparent locomotor effects^133,169^, though others observed an enhanced locomotion^170,171^. On the other hand, changes on anxiety behaviors were described in rats^171^, but not in mice^169^ (Table 3).

Social behaviors were shown to be affected by postnatal exposure to VPA in rats and mice^168,170,171^, though others could not replicate some of these findings^169^. In the same conditions, higher levels of social aggression were found^169^ (Table 3).

Several cognitive aspects appeared to be affected in these models, including deficits in spatial^133^ and reversal learning^169^, accompanied by enhanced cue-dependent reward learning^168^. Additionally, other studies described longer latencies in a passive avoidance paradigm in mice^133^, while others failed to reproduce some of these effects in rats^171^ (Table 3).

## Neural mechanisms engaged in the rodent models of early-life exposure

In the last decade, many preclinical studies have contributed to our understanding of the possible molecular, cellular and circuit mechanisms implicated in the neurodevelopmental vulnerability to psychiatric conditions. A main concept emerging from those studies is that the ontogenetic occurrence of unique developmental events defines critical periods of plasticity with a maximal sensitivity to environmental functional demands^14–16,18^. Thus, depending on the period when the environmental challenge/perturbation takes place, the long-lasting impact that it will have on the mature brain. In this section, we explore maladaptive neural mechanisms that have been implicated in the behavioral outcomes of the analyzed rodent models, paying special attention to brain regions and neural mechanisms that could be similarly altered across the different models.

### Developmental role of 5-HT and Prefrontal circuits

Perturbation of the 5-HT neural signaling during early-life has been long associated with developmental origins of several psychiatric conditions, including anxiety, depression, and ASD^43,44,172–176^. In rodents, activation of 5-HT receptors in different brain regions has been implicated in long-term emotional alterations. Thus, in the early-life stress model of maternal separation, an enhanced adult 5-HT2A/C-mediated prefrontal function was found^62^, while the early postnatal pharmacological blockade of these receptors prevented the emergence of the anxiety phenotype in the same model^63^. Interestingly, the expression of 5-HT2A/C receptors appeared to be substantially modified by prenatal stress but not after maternal separation^55,63^. Emotional alterations produced by the postnatal exposure to SSRIs also appear to be mediated, at least in part, by 5-HT2A/C receptors. That is, the exposure to 5-HT2A and 5-HT2C antagonists during the early postnatal period prevents the anxiety and depressive-like phenotypes induced by fluoxetine, while the treatment in the same period with agonists of the same receptors produces anxiety^104^.

Other 5-HT receptors such as the 5-HT1A and 5-HT7 were also implicated in the behavioral consequences of early postnatal SSRIs. That is, some of the adult emotional effects induced by postnatal fluoxetine were found to be enhanced by the co-treatment with a 5-HT1A receptor agonist^116^. More recently, the prefrontal 5-HT7 receptors have been shown to have a crucial developmental role in the emergence of anxiety and depressive-like symptoms in the model of postnatal fluoxetine^112^. Additionally, increased stimulation of 5-HT1A receptors during early postnatal life was also shown to mimic deficits produced by early SSRIs on adult social interactions of relevance to ASD^128^. On the other hand, in the VPA model, activation of 5-HT1A receptors improves some of the social and cognitive deficits produced in that model^166^.

**Fig. 1:**
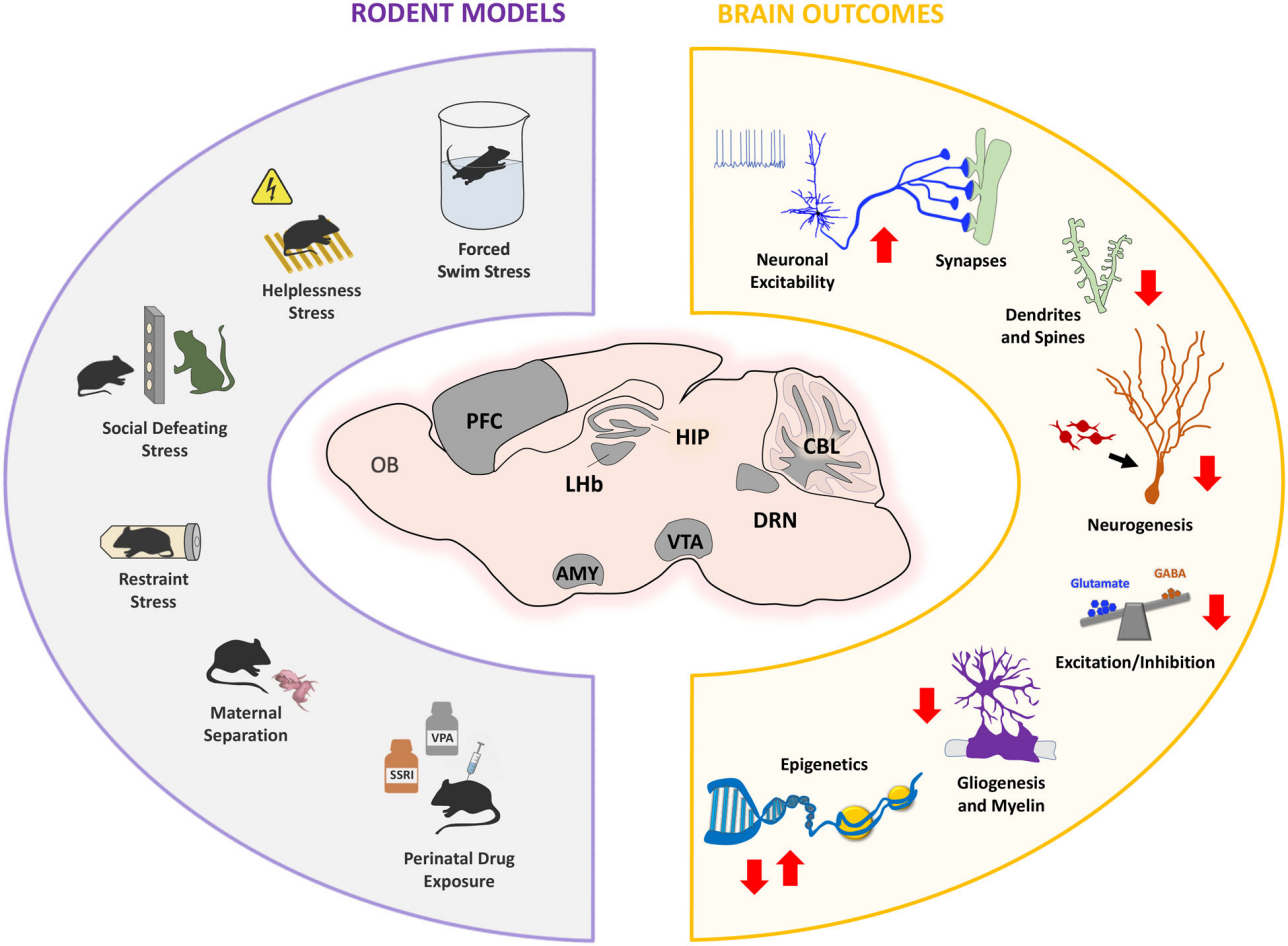
Schematic overview of some of the main effects reported after either stress, SSRI or VPA exposures during gestation and/or early postnatal life, on different brain regions and neurodevelopmental processes. Different rodent models of exposure to stress (forced swim, helplessness, social defeat, restraint, maternal separation) and to chemical substances (SSRIs, VPA) are shown. Main brain regions affected in these models are indicated (Olfactory bulb OB, Prefrontal cortex PFC, Lateral habenula LHb, Amygdala AMY, Hippocampus HIP, Ventral tegmental area VTA, Dorsal raphe nucleus DRN, Cerebellum CBL), together with summarized effects on neurodevelopmental molecular, cellular, and circuit mechanisms. These include hyperexcitability of glutamate neurons, exuberant glutamate synaptogenesis, decreases in dendrite and spine remodeling, decreased neurogenesis, reduction of the balance of glutamate/GABA transmission, reduced gliogenesis and myelination, and multiple changes in epigenetic control of gene expression.

Accumulating evidence indicates a primordial role of the prefrontal cortex (PFC) in these neurodevelopmental mechanisms. Thus, disruptions of developmental processes in the PFC such as circuit formation/refinement, synaptic connectivity, and oligodendrogenesis/myelinization, have been directly linked to the early vulnerability to stress-related and emotional alterations (Fig. 1). Prenatal stress has been reported to produce a decrease in spine density in mPFC pyramidal neurons^48^. A similar reduction was described in layer 2–3 mPFC pyramidal cells after postnatal exposure to fluoxetine, accompanied by altered excitability of this neuronal population and exuberant dendritic branching^106,115^. Functional imaging analysis in trumpet-tailed rats subjected to maternal separation stress has shown a global decrease in brain activity in PFC circuits likely engaged in prefrontal-limbic control^177^. Besides, decreases in the excitability of PFC pyramidal neurons have been recently described after maternal separation^178^. Consistent findings were described when analyzing the expression of activity-related immediate early genes in the PFC in the same model, accompanied by a precocious oligodendrocyte differentiation, and hypo-myelination^67,179,180^. In agreement with this, postnatal exposure to the SSRI citalopram, but not the prenatal treatment, was shown to alter the oligodendrocyte morphology and the callosal connectivity^126^.

Recent studies have shown a synaptic hyper-connectivity of corticolimbic circuits such as the PFC-to-dorsal raphe nucleus (DRN) one, after postnatal exposure to fluoxetine during the early postnatal period^111^. Furthermore, these changes were found to be mediated by the developmental 5-HT signaling through the 5-HT7 receptors in the PFC^112^. Importantly, reciprocal circuits connect the PFC to DRN 5-HT neurons, and these pathways have a crucial role in controlling stress-coping strategies and emotional responses throughout life^111,181,182^.

Direct inhibition of the activity of DRN 5-HT neurons using chemogenetic tools has been shown to prevent the emergence of the emotional alterations produced by postnatal fluoxetine^107^. On the other hand, the early-life treatment with another SSRI (i.e., citalopram), was shown to reduce the 5-HT synthesis and its forebrain release^123^. Additionally, maternal separation reduces the firing activity of DRN 5-HT neurons^183^. Interestingly, gestational exposure to VPA was reported to delay the migration and differentiation of developing DRN 5-HT neurons^184^.

Altogether, this evidence indicates a complex role of the developing 5-HT system and its targeted neural circuits, in the detrimental emotional effects produced by early-life exposures.

### **Other neural circuits implicated**

Other studies have implicated other brain structures such as the ventral tegmental area (VTA) and lateral habenula (LHb), in the emotional vulnerability during early-life^64,82^ (Fig. 1). There, genetic invalidation of an early transcriptional program in the VTA was sufficient to replicate the life-long susceptibility to stress observed after maternal separation^64^. Furthermore, a recent study using the same model showed enhancements in the excitability of VTA dopamine neurons and their excitatory synaptic inputs^185^. In another study, researchers showed that limiting the hyperactivity of LHb output neurons relieves the depressive-like symptoms caused by maternal separation^82^. Interestingly, both VTA and LHb neurons are interconnected, and receive dense 5-HT afferents^186^, and in turn, send projections back to DRN neurons^187,188^.

The hippocampus appeared to be more robustly affected in preclinical models using either gestational or precocious early postnatal exposures. Thus, alterations in neurogenesis were found among the different preclinical models^52,59,81,110,189^ (Fig. 1). Specifically, changes in the survival of newborn cells in the dentate gyrus of the hippocampus^52,59,81,189^ and alterations in the expression of neurogenic modulators such as brain-derived neurotrophic factor (BDNF)^52,110^, have been consistently reported after either prenatal or early postnatal stress protocols. Similarly, effects on the hippocampal neurogenesis and BDNF expression levels were also described after maternal separation, SSRI and VPA postnatal exposures^108,110,113,189–191^. Interestingly, postnatal SSRI exposure reverses alterations on hippocampal neurogenesis produced by prenatal maternal stress^189^.

Alterations in the synaptic morphology and function of the hippocampus were implicated in the models of prenatal exposures to VPA and stress^53,135,159,192^. Interestingly, in the stress model, postnatal treatment with SSRIs restores the normal density of dendritic spines and glutamate synapses^119^. In this last model, altered formation and maintenance of the hippocampal perineuronal net has been recently shown after SSRI exposure^193^. Furthermore, an altered synaptic development of glutamate/GABA transmission systems has been also described in the PFC, after gestational exposures to SSRIs or VPA^48,99,135,144,159,192^ (Fig. 1). In addition, these changes were accompanied by a delayed neuronal migration followed by a precocious neuronal maturation, and long-standing modifications in cortical thickness^137,144,168^. Interestingly, cortical modifications on dendritic spines and neuronal morphology were also observed after postnatal exposure to VPA^169^ (Fig. 1), likely reflecting the protracted developmental period of frontocortical circuits. Lastly, at the system level, the early-life stress experience of maternal separation was shown to perturb the communication within the network engaging hippocampal and PFC circuits^194^.

Other studies point out to the amygdala as a key neural substrate involved in the effects of maternal separation and *in utero* VPA exposure (Fig. 1). These investigations showed a reduced functional connectivity between the basolateral amygdaloid nucleus (BLA) and the PFC after maternal separation^195^, in agreement with previous imaging findings in depressive patients^196,197^. On the other hand, enhancements of the neuronal excitability and glutamate transmission in the amygdala have been reported in the VPA model^142,153^. Besides, similar findings were described in the same model in frontocortical circuits^158,165,198,199^ (Fig. 1). Interestingly, treatment with a metabotropic glutamate receptor 5 (mGluR5) antagonist rescued some of the social behavioral deficits observed in the VPA model^138^. Additionally, changes on GABAergic neurochemical markers and modifications on the neuronal/glial cytoarchitecture, have been also reported in the amygdala and cerebellum, after prenatal exposure to VPA^135,156,200^.

### **Epigenetic mechanisms**

Other lines of evidence indicate an important role for epigenetic control of gene expression in the neurodevelopmental mechanisms at play in these preclinical models (Fig. 1). For instance, a transcriptional disruption of genes involved in developmental and immune gene networks has been described in the amygdala after VPA exposure^157^. In other studies, researchers found that acetylation regulation of hippocampal gene expression by the histone deacetylase (HDAC) 4 is crucially involved in the adult emotional alterations caused by postnatal SSRIs^105^. Consistent with its relevance, a decreased expression of several members of the HDAC family was reported in frontocortical regions including the PFC of maternally-separated rats and mice^180,201^. Furthermore, in one of these studies the authors suggested that this mechanism could be a major contributor to the susceptibility/resilience to early-life stress, and the subsequent efficacy to antidepressant treatments^201^. *In utero* effects of VPA have been directly linked to its actions on the activity of HDACs. Thus, exposure to the HDAC inhibitor trichostatin A, can phenocopy many of the social behavioral deficits observed in the VPA model^145^. Consistently, the prenatal treatment with valpromide, a VPA analog lacking the HDAC’s inhibitory activity, failed to reproduce the social interaction deficits^137^.

Methylation of promoter regions has been also implicated in the effects of SSRIs during pregnancy and lactation^98,109^. These investigations showed long-lasting changes in methylation levels of multiple genes in the hippocampus and cortex, including the *Bdnf* gene^98,109^. Interestingly, chromatin remodeling and increased methylation levels in the PFC were reported, after either gestational stress^48^ or VPA exposure^202^. Lastly, the transgenerational epigenetic inheritance of VPA-induced imbalance in excitatory/inhibitory transmission in the frontal cortex has been recently demonstrated^203^, illustrating the rather complex repertoire of neurodevelopmental mechanisms that could be engaged in the different preclinical models of exposure.

## Translational Aspects and Concluding Remarks

Rodent models have been very useful to investigate the mechanisms implicated in the developmental vulnerability to psychiatric disorders. Thus, preclinical research surveyed in this review allows the identification of neural substrates and neurobiological mechanisms impacted by early-life environmental exposures. Importantly, many of these findings may directly relate to what occurs in the complex human psychopathology. However, other biological processes linked to gene regulation and its possible interaction to environmental conditions, is growingly emerging as crucial actors involved in neuropsychiatric disorders. For instance, investigations in rodents with reduced 5-HT synthesis have shown a differential impact on the DRN 5-HT system of maternal separation^204^, while reduction of the 5-HT transporter, SERT, leads to an enhanced anhedonia under similar stress conditions^205^. Furthermore, combination of stress exposure and postnatal SSRI treatment in dams alters affective susceptibility of the offspring in a SERT-dependent manner^206^. Conversely, behavioral alterations produced by *in utero* VPA exposure were not affected by the presence of SERT^155^. On the other hand, rats with reduced SERT expression, and a previous history of maternal separation, showed improvements in stress-coping responses^207^. Interestingly, *in utero* exposure to citalopram mitigates the detrimental effects of maternal stress on the fetal forebrain development, and these changes are thought to be mediated by normalizing brain 5-HT levels^88^.

Human studies have shown that gestational exposure to SSRIs is associated with adverse neonatal outcomes that can be moderated by the *SERT* promoter polymorphism of the infants^20^. Moreover, methylation of the *SERT* promoter can influence the soothability of infants that had exposition to SSRIs during the gestation^208^. Additionally, the hyperserotonemia and several stereotypical behavioral outcomes present in ASD patients can be replicated in mice that express a hyperfunctional SERT variant^209,210^.

A critical point to be considered when analyzing the risk of exposure to SSRIs in pregnant women is the psychopathological state of the future mother. Evidence indicates that SSRI treatment in depressed mothers can prevent the modifications in brain’s connectivity produced in newborns that are exposed to unmedicated depressive mothers^211^. In addition, other studies have shown that adverse effects produced by prenatal maternal depression on infant’s problematic temperament can be amplified by a concurrent prenatal traumatic stress^212^.

In the case of VPA, human evidence supports only a poor interaction between the effects of prenatal exposure to VPA and maternal mental health. Thus, a very recent nationwide population-based analysis has shown that *in utero* exposure to VPA is associated with an increased risk of neurodevelopmental mental and behavioral disorders in exposed children^213^. Moreover, these effects were stronger when exposures occurred during the second and third trimesters of pregnancy. However, these findings were not affected by the mother’s mental health^213^.

Future investigations need to consider neurodevelopmental regulatory mechanisms as well as other biological factors, such as the neuroinflammatory and hormonal contexts, that could influence the brain’s homeostatic capacity to mitigate early-life environmental perturbations. Altogether, these further considerations will improve understanding of how developmental maladaptive mechanisms could increase the risk of vulnerability to mental disorders.
